# Natural health products that inhibit angiogenesis: a potential source for investigational new agents to treat cancer—Part 2

**DOI:** 10.3747/co.v13i3.88

**Published:** 2006-06

**Authors:** S.M. Sagar, D. Yance, R.K. Wong

**Affiliations:** * Juravinski Cancer Centre and McMaster University (Department of Medicine), Hamilton, Ontario; † Center for Natural Healing, Ashland, Oregon, U.S.A

**Keywords:** Angiogenesis, anti-angiogenic, natural health products, herbal medicine, anticancer, clinical trials, integrative, molecular biology

## Abstract

The herbalist has access to hundreds of years of observational data on the anticancer activity of many herbs. Laboratory studies are expanding the clinical knowledge that is already documented in traditional texts. The herbs that are traditionally used for anti-cancer treatment and that are anti-angiogenic through multiple interdependent processes (including effects on gene expression, signal processing, and enzyme activities) include *Artemisia annua* (Chinese wormwood), *Viscum album* (European mistletoe), *Curcuma longa* (curcumin), *Scutellaria baicalensis* (Chinese skullcap), resveratrol and proanthocyanidin (grape seed extract), *Magnolia officinalis* (Chinese magnolia tree), *Camellia sinensis* (green tea), *Ginkgo biloba,* quercetin, *Poria cocos, Zingiber officinalis* (ginger), Panax ginseng, *Rabdosia rubescens* hora (Rabdosia), and Chinese destagnation herbs. Natural health products target molecular pathways other than angiogenesis, including epidermal growth factor receptor, the *HER2*/*neu* gene, the cyclo-oxygenase-2 enzyme, the nuclear factor kappa-B transcription factor, the protein kinases, the Bcl-2 protein, and coagulation pathways. Quality assurance of appropriate extracts is essential prior to embarking upon clinical trials. More data are required on dose–response, appropriate combinations, and potential toxicities. Given the multiple effects of these agents, their future use for cancer therapy probably lies in synergistic combinations. During active cancer therapy they should generally be evaluated in combination with chemotherapy and radiation. In this role, they act as modifiers of biologic response or as adaptogens, potentially enhancing the efficacy of the conventional therapies or reducing toxicity. Their effectiveness may be increased when multiple agents are used in optimal combinations. New designs for trials to demonstrate activity in human subjects are required. Although controlled trials may be preferable, smaller studies with appropriate endpoints and surrogate markers for anti-angiogenic response could help to prioritize agents for larger, resource-intensive phase iii trials.

## 1. INTRODUCTION

The biochemical signalling pathways of angiogenesis form a complex, interconnected web. Inhibition of one part of the web may result in compensation through another pathway. Because botanicals contain a variety of organic chemical complexes, they usually act on multiple targets. A potential advantage of phytochemicals is that they may act through multiple pathways and reduce the development of resistance by cancer cells. This model of pharmacognosy recognizes the advantage of administering the whole plant product to maximize activity. Over-extraction of a specific chemical constituent may remove this therapeutic gain. The challenge for modern pharmacognosy is to ensure that the optimum mixture of chemical constituents is maintained when a product is purified. Usually, such assurance will require a combination of chemical and biologic assays.

The additional anticancer properties of some anti-angiogenic botanicals are briefly discussed here. Their properties may affect various biochemical pathways that indirectly influence angiogenesis. Traditional practice has been to combine multiple natural health products, and scientifically, such combination may provide a therapeutic advantage.

## 2. MULTISTEP ACTIVITY OF PHYTOCHEMICAL COMPLEXES DERIVED FROM HERBS

### 2.1 Targeting Alternative Angiogenesis Pathways

The adipocytokines—polypeptides produced by adipocytes—have autocrine, paracrine, and endocrine activities, and are associated with obesity, hyperinsulinemia, and chronic vascular disease as well as with the development of cancer 1. The adipocytokines include vascular endothelial growth factor (vegf), hepatocyte growth factor (hgf), leptin, tumour necrosis factor alpha (tnfα), heparin-binding epidermal growth factor, insulin-like growth factor, and interleukin-6 (IL-6). All can promote angiogenesis.

Curcumin (from turmeric) and epigallocatechin-3 gallate (egcg, from green tea) can inhibit aminopeptidase-N (CD13), a member of the matrix metalloproteinase family that is implicated in the angiogenic switch process 2–4. Curcumin and egcg can also interfere with the expression of vegf by suppressing a series of activities that promote angiogenesis. These angiogenic pathways include production of transforming growth factor beta (tgfβ), amplification of cyclooxygenase-2 (cox-2) and epidermal growth factor receptor (egfr), aberrant expression of *src*, and amplification of nuclear factor kappa-B (nf-κb) signalling. Curcumin, grape seed extract, and green tea constituents may also interfere with endothelial cell function by inhibiting the engagement of specific integrins 5,6. These phytochemicals interact at multiple levels to suppress the inflammatory, hyperproliferative and transformative processes that constitute carcinogenesis.

### 2.2 Targeting EGFR (*HER1*)

In many human tumours, egfr is overexpressed. Such overexpression is associated with more aggressive disease, relative resistance to cytotoxic chemotherapy, and a poorer prognosis. Activity of egfr induces angiogenesis 7. Blockade of egfr reduces angiogenesis and cell proliferation 8. Monoclonal antibodies have been developed to block the receptor or the linked intracellular signalling system 9–15.

Epidermal growth factor (egf) stimulates urokinase-type plasminogen activator (upa) expression, which can promote angiogenesis. Genistein (an isoflavone constituent of soy) and curcumin (a constituent of turmeric) both inhibit the effects of egf 16. In cell cultures, genistein and curcumin inhibit egf-stimulated urokinase production and phosphorylation of egfr. Both botanicals also inhibit protein tyrosine kinases, which could stimulate the enhancement of upa levels induced by tgfβ 17.

Other natural health products that can block activity of egfr include resveratrol 18 and quercetin 19–21.

### 2.3 Targeting *HER2*/*neu*

The *HER2*/*neu* gene (formerly known as c-*erbB*-2) is amplified in more than 30% of patients with breast cancer. It is linked to highly aggressive tumours with a poorer prognosis. Overexpression of *HER2* is also seen in a significant proportion of patients with other cancer types, including non-small-cell lung cancer, ovarian cancer, prostate cancer, and gastric cancer, in which it may predict a worse outcome 22–26. Amplification of the *HER2* gene correlates with higher levels of angiogenesis 27.

Herceptin (Genentech, San Francisco, CA, U.S.A.) is a drug that inhibits *HER2*/*neu*. It is usually administered adjunctively with cytotoxic chemotherapy. The activity of Herceptin may be further enhanced by oleic acid 28. Emodin, a natural constituent of *Polygonum multiflorum* and aloe, inhibits *HER2*/*neu* expression and is toxic against cancer cells, but nontoxic for normal cells 29.

### 2.4 Targeting Inflammatory Pathways: COX-2 and NF-κB

Prostaglandins are autacoids derived from arachidonic acid via the cox enzymes. They include prostacyclin, thromboxane, and prostaglandin E types 1–3. A role for arachidonic acid–derived prostaglandins in the process of angiogenesis is now established through *in vitro* assays. Prostaglandin E2 is a potent inducer of angiogenesis. A correlation exists between cox-2 expression and angiogenesis 30. Neovascularization is blocked by cox-2 antagonists 31–36.

The cox-2 and lipoxygenase (lox-5) products of omega-6 fatty acid metabolism may exert stimulatory effects on cancer progression including angiogenesis. The omega-3 fatty acids and some pharmacologic inhibitors of eicosanoid biosynthesis antagonize these effects 37–41. Large amounts of omega-3 fatty acids [eicosapentaenoic acid (epa) and docosahexaenoic acid] are found in cold-water fish oils. Liquorice contains glycyrrhizic acid and polyphenols that inhibit cox-2, lox-5, and protein kinase C (pkc) and also downregulate egf 42.

The nf-κb family consists of closely related protein dimers that bind to a common sequence motif in dna called the κb site. The nf-κb inducible transcription factor is increased in tissue inflammation, cell proliferation, and cancers. Nuclear factor kappa B induces overactivation of the cox enzymes and is associated with increased angiogenesis 43–46.

The cox enzymes are expressed in most normal tissues. The cox-1 enzyme synthesizes non-inflammatory prostaglandins such as prostaglandin E1. In contrast, cox-2 is amplified as part of the inflammatory response and produces prostaglandins such as prostaglandin E2, which may induce uncontrolled cell proliferation and carcinogenesis.

Nuclear factor kappa B may be amplified by growth factors, including tgfβ and basic fibroblast growth factor. Besides nf-κb, other transcription factors, such as activator protein-1 (ap-1) and IL-6, can stimulate cox-2 transcription. Activator protein-1 also promotes the metastatic phase of tumour cells.

Angiogenesis mediated by cox-2 also has a role in the progression of pre-neoplastic lesions to the invasive phenotype 47–50. Conventional cancer therapies—such as radiation, surgery, and chemotherapy—may induce cox-2 amplification as part of the inflammatory response 51. The significance of this induction is unclear, but it could hypothetically reduce therapeutic gain.

Several phytochemical derivatives are potent inhibitors of nf-κb. These include resveratrol, piceatannol, curcumin, egcg, 6-gingerol (ginger), ursolic acid (holy basil), and ginseng 52–56. Many botanical cox-2 inhibiting agents block the amplified activity of the transcription factor nf-κb without affecting its normal function.

A variety of natural health products can specifically inhibit the cox-2 enzyme and could play a role in reducing tissue toxicity and improving tumour control when used alongside therapies such as radiotherapy, chemotherapy, and surgery (Table I) 57. A botanical that protects an organism from the adverse effects of an intervention is termed an adaptogen. Panax ginseng and curcumin are adaptogens that inhibit cox-2 and that have anti-angiogenic activity derived through the inactivation of nf-κb 55,58–61

### 2.5 Targeting Protein Kinases

Oncogenes that encode protein kinases may contribute to the development of cancer. In normal cells, protein kinases are involved in signals between the cell membrane and the nucleus, regulating progression through the cell cycle. Protein kinases control these processes by activating other messenger proteins that can influence cell proliferation.

Mutated kinase genes have been found in a number of malignancies, including chronic myelogenous leukemia and breast and bladder cancers. The mutated kinases can contribute to the development of cancer. In many tumour cells, protein kinases are permanently turned on, forcing the cell into constant division. Examples of abnormal kinases are the *abl, src,* and cyclin-dependent kinases. The kinases may be amplified or permanently switched on by mutations in the control regions of their genes. A commonly overproduced kinase in cancer is egfr.

Numerous phytochemicals are reported to interfere with cell signalling and may reverse the adverse effects of protein kinase overactivity. Some botanicals with cox-2 inhibitory activity target the intracellular signalling molecules 62,63. Inhibition of specific protein kinases suppresses angiogenesis 64–69.

Carnosol and ursolic acid are compounds found in *Ocimum sanctum* (holy basil) and *Rosmarinus officinalis* (rosemary) 70. They inhibit the activity of the tyrosine kinases and ornithine decarboxylase 71. Carnosol also reduces nf-κb 72 and the anti-apoptotic protein Bcl-2 73. Genistein and daidzein (isoflavones found in soy) are specific inhibitors of tyrosine kinases 74.

Many phytochemicals appear to selectively react with the regulatory centre of pkc. Curcumin, vitamin E, green tea (catechins), resveratrol, *Ganoderma lucidum,* and liquorice can inhibit pkc activity 42,75–77

### 2.6 Targeting the Bcl-2 Protein

The signalling protein Bcl-2 plays a key role in the process of controlled cell death called apoptosis, which is necessary to eliminate aged or damaged cells. The Bcl-2 protein is normally found in the mitochondrial membrane, where it regulates the release of cytochrome C. The latter protein can trigger a series of enzymes (caspases) that lead to cell death 78–81. High Bcl-2 levels are associated with most types of human cancer and block the release of cytochrome C. They appear to be a contributor to both inherent and acquired resistance to anticancer treatments. The *BCL2* and *TP53* genes regulate vegf-mediated angiogenesis 82

Curcumin and green tea extract inhibit *BCL2* expression 83–85. *Scutellaria baicalensis* contains the phenolic compounds baicalin, baicalein, wogenin, and oroxylin. These constituents inhibit *BCL2* overexpression, plus *COX2* gene expression and nf-κb activation 86,87. Hibiscus protocatechuic acid is a phenolic compound isolated from the dried flower of *Hibiscus sabdariffa* L.; it inhibits Bcl-2 activity 88,89. Other inhibitors of Bcl-2 include epa from fish oil 90, a lectin extract of *Viscum album* (mistletoe) 91, 6-gingerol 92, grape seed extract 93, echinocystic acid (a triterpene found in ginseng and other Asian herbs) 94,95, parthenolide (a sesquiterpene lactone found in feverfew) 96, and beta-lapachone (a quinone obtained from the bark of the lapacho tree) 97–99.

### 2.7 Targeting Coagulation Pathways Associated with Angiogenesis

In some clinical trials, anticoagulation drugs have been associated with a reduction in metastases 100–102. In Chinese medicine, destagnation herbs are traditionally thought to overcome blockages of *qi* and blood. Laboratory evidence now suggests that these herbs may have anti-angiogenic and anticoagulation properties 103–105. A randomized placebo-controlled trial showed that the addition of “destagnation” herbs (including *Salvia miltiorrhiza* and *Angelica sinensis*) to radiotherapy doubled both the local control and the survival rate in patients with nasopharyngeal cancer 106.

## 3. CONCLUSION

Angiogenesis involves multiple interdependent processes operating at the molecular level. These include gene expression, signal processing, and enzyme activities. Most anti-angiogenic natural health products block new vessel formation at multiple levels.

Lack of standardization of screening assays may be an obstacle to defining the most effective products for clinical use. Over-extraction of constituents may negate some of the potential synergy. Quality assurance of appropriate extracts is essential before embarking upon clinical trials.

Most studies of anti-angiogenic activity are based on *in vitro* or animal work that cannot be readily extrapolated to humans. Phase i and ii studies are required to determine the potential of these substances to improve cytotoxic therapies. Mainly preclinical data exist for most of the naturally derived anti-angiogenic agents. However, because anti-angiogenic agents are mainly cytostatic in nature, the usual paradigm for anticancer drug development, in which tumour response in phase ii trials prompts further development, is not always appropriate. More data are required on dose–response, appropriate combinations, and potential toxicities. Given the multiple effects of these agents, their future use for cancer therapy probably lies in synergistic combinations. They may be evaluated alone for the prevention of cancer recurrence following definitive treatment.

To be suitable for long-term chronic use, these agents should possess minimal toxicity and should be orally administered. However, angiogenesis is also essential for healing of injuries. Most compounds that inhibit tumour angiogenesis are likely to inhibit physiologic angiogenesis, leading to potential side effects such as ulceration and bleeding. Studies are required to determine features that distinguish tumour vessels from normal vessels so that a therapeutic gain can be achieved. Some of the differences have already been described, but the doses and scheduling of anti-angiogenic agents appropriate to achieving the optimum therapeutic gain are unclear.

During active cancer therapy anti-angiogenic agents should generally be evaluated in combination with chemotherapy and radiation. In that role, they act as modifiers of the biologic response and as adaptogens, potentially enhancing the efficacy and reducing toxicity of conventional therapies. The combination of diversity in angiogenic factor expression and different phenotypes of endothelial cells within various tumours is a major challenge for the development of effective anti-angiogenic regimens 107,108. Effectiveness may be increased when multiple agents are used in optimal combinations.

Surrogate markers, such as angiogenic cytokines, are necessary to predict anti-angiogenic response 109. Circulating levels of fibroblast growth factor–2, vegf, vascular cell adhesion molecule–1, endothelial inter-cellular adhesion molecule–1, insulin-like growth factor–1, and cytokines such as interleukin-8 may correlate with tumour angiogenesis 110–114. In addition, circulating endothelial cells and their progenitors may be a more reliable marker of response to anti-angiogenic therapies 115,116. Non-invasive functional imaging, such as positron emission tomography and functional magnetic resonance imaging, may play a role 117.

Current laboratory evidence suggests a useful role for natural health products in the treatment of cancer. The input of an herbalist, an oncologist, a laboratory scientist, and a clinical trials methodologist to the research effort is essential to distil the wealth of traditional knowledge into a modern framework that can be evaluated scientifically. Information on traditional dose levels is important for designing initial phase i clinical trials for safety and maximum tolerated dose (Table II). However, the traditional model of pharmacognosy may not necessarily use the highest dose. Establishing the maximum tolerated dose in a phase i study may not always be appropriate. Instead, determination of the biologically active dose that may possess less toxicity may be more relevant. Combinations of whole herbs or constituent phytochemicals at lower doses may be important. In addition, a longer period of exposure to the natural health product may be more effective than a short exposure to the highest possible dose level. New designs for trials to demonstrate activity in human subjects are required.

Although controlled trials might be preferred, smaller studies with appropriate endpoints and surrogate markers for anti-angiogenic response could help to prioritize agents for the larger, resource-intensive phase iii trials. Because most of the agents are expected to be cytostatic, it is inappropriate to require the standard criteria of measured tumour response. On the other hand, simply confirming stable disease may be misleading. More research on surrogate markers of anti-angiogenic response is obviously necessary before resources can be directed to large-scale clinical trials (Table III).

The further development of natural health products for clinical trials will require a team effort between academic centres, government, and industry. Appropriate financial support and market protection will be necessary to encourage this activity. New ways of supporting evidence-based innovation for natural health products are necessary, without necessarily separating and isolating all the constituents of a natural health product simply to allow patent registration. Commercial protection for companies that provide quality assurance and clinical trials evidence may be necessary. Another model includes government support for evaluation, with some profit being returned to the government when a product is commercialized. Part of the funding for clinical studies of the AE-941 shark cartilage derivative (Neovastat: Æterna Zentaris, Quebec, QC, Canada) came from Technology Partnerships Canada, a research support program of the federal government of Canada 118.

Teamwork between the oncologist, the herbalist, the laboratory scientist, and the research methodologist is important for studying anti-angiogenic herbs and other natural health products. As clinical trials introduce these products into the clinic, more definitive evidence of efficacy will be provided and more cancer patients may potentially experience improved outcomes.

## Figures and Tables

**Table I:** Natural health products that inhibit cyclo-oxygenase-2 activity 57

Ginger
Aloe vera
egcg/green tea
Resveratrol
Liquorice
Garlic
Chinese skullcap
Bilberry
Grape seed extract proanthocyanidins
Panax ginseng
Milk thistle
Fish oils: omega-3 fatty acids (eicosapentaenoic acid, docosahexaenoic acid)
Green-lipped mussel
Antioxidants (vitamins A, C, E; Se, Zn; carotenoids, flavonoids, coenzyme Q10, *N*-acetylcysteine, lipoic acid)
Boswellia
Bromelain
Curcumin
Quercetin

**Table II:** Dose ranges of some phytochemicals used by an herbalist for angiogenesis inhibition

*Herb/phytoceutical*	*Preventive dose*^a^	*Cancer adjuvant dose*^a^
Turmeric (95% curcumin)	500–1000 mg daily	1000–2500 mg 3 times daily
Green tea (95% phenols; 50% EGCG)	200–500 mg daily	1000–1200 mg 3 times daily
Grape seed extract (95% proanthocyanidin)	100–200 mg daily	600–1000 mg daily
Japanese knotweed (20% resveratrol)	30–50 mg daily	300–500 mg daily
Quercetin with bromelain	500–1500 mg daily	500–1000 mg 3 times daily
Holy basil and rosemary (2.37% and 1.5% ursolic acid)	10–20 mg day	10–20 mg 3 times daily
Silibinin (80% silymarin)	200 mg daily	Up to 2000 mg 3 times daily

a Note that these dose ranges have not all been evaluated in clinical pharmacokinetic studies and are not approved by the U.S. Food and Drug Administration or Health Canada at this stage. The Natural Health Products Directorate of Health Canada is in the process of registering quality, efficacy, and dosing data for natural health products).

egcg = epigallocatechin-3 gallate.

**TABLE III:** Potential surrogate blood tests for monitoring angiogenesis and its response to therapies 109–116

Circulating vascular molecules
Vascular endothelial growth factor
Fibroblast growth factor–2
Interleukin-8
Insulin-like growth factor–1
Vascular adhesion molecule–1
Endothelial intercellular adhesion molecule
Matrix metalloproteinase–9
Circulating cells
Circulating endothelial cells
Circulating endothelial cell progenitors (CD34+ peripheral blood mononuclear cells)

## References

[1] Rose DP, Komninou D, Stephenson GD (2004). Obesity, adipocytokines, and insulin resistance in breast cancer. Obes Rev.

[2] Shim JS, Kim JH, Cho HY (2003). Irreversible inhibition of CD13/aminopeptidase N by the antiangiogenic agent curcumin. Chem Biol.

[3] Tang FY, Nguyen N, Meydani M (2003). Green tea catechins inhibit vegf-induced angiogenesis *in vitro* through suppression of ve-cadherin phosphorylation and inactivation of *akt* molecule. Int J Cancer.

[4] Kojima–Yuasa A, Hua JJ, Kennedy DO, Matsui–Yuasa I (2003). Green tea extract inhibits angiogenesis of human umbilical vein endothelial cells through reduction of expression of vegf receptors. Life Sci.

[5] Singh RP, Tyagi AK, Dhanalakshmi S, Agarwal R, Agarwal C (2004). Grape seed extract inhibits advanced human prostate tumor growth and angiogenesis and upregulates insulin-like growth factor binding protein-3. Int J Cancer.

[6] Khanna S, Roy S, Bagchi D, Bagchi M, Sen CK (2001). Upregulation of oxidant-induced vegf expression in cultured keratinocytes by a grape seed proanthocyanidin extract. Free Radic Biol Med.

[7] Casanova ML, Larcher F, Casanova B (2002). A critical role for *ras-*mediated epidermal growth factor receptor–dependent angiogenesis in mouse skin carcinogenesis. Cancer Res.

[8] Wu JL, Abe T, Inoue R, Fujiki M, Kobayashi H (2004). IκBαM suppresses angiogenesis and tumorigenesis promoted by a constitutively active mutant egfr in human glioma cells. Neurol Res.

[9] Salomon DS, Brandt R, Ciardiello F, Normanno N (1995). Epidermal growth factor-related peptides and their receptors in human malignancies. Crit Rev Oncol Hematol.

[10] Nicholson RI, Gee JM, Harper ME (2001). egfr and cancer prognosis. Eur J Cancer.

[11] Kawamoto T, Sato JD, Le A, Polikoff J, Sato GH, Mendelsohn J (1983). Growth stimulation of A431 cells by epidermal growth factor: identification of high-affinity receptors for epidermal growth factor by an anti-receptor monoclonal antibody. Proc Natl Acad Sci U S A.

[12] Masui H, Kawamoto T, Sato JD, Wolf B, Sato G, Mendelsohn J (1984). Growth inhibition of human tumor cells in athymic mice by anti-epidermal growth factor receptor monoclonal antibodies. Cancer Res.

[13] Sato JD, Kawamoto T, Le AD, Mendelsohn J, Polikoff J, Sato GH (1983). Biological effects *in vitro* of monoclonal antibodies to human epidermal growth factor receptors. Mol Biol Med.

[14] Woodburn JR (1999). The epidermal growth factor receptor and its inhibition in cancer therapy. Pharmacol Ther.

[15] Noonberg SB, Benz CC (2000). Tyrosine kinase inhibitors targeted to the epidermal growth factor receptor subfamily: role as anticancer agents. Drugs.

[16] Smith PC, Santibanez JF, Morales JP, Martinez J (2004). Epidermal growth factor stimulates urokinase-type plasminogen activator expression in human gingival fibroblasts: possible modulation by genistein and curcumin. J Periodontal Res.

[17] Shao ZM, Wu J, Shen ZZ, Barsky SH (1998). Genistein inhibits both constitutive and egf-stimulated invasion in er-negative human breast cancer cell lines. Anticancer Res.

[18] Igura K, Ohta T, Kuroda Y, Kaji K (2001). Resveratrol and quercetin inhibit angiogenesis *in vitro.*. Cancer Lett.

[19] Banerjee T, Van der Vliet A, Ziboh VA (2002). Down regulation of cox-2 and inos by amentoflavone and quercetin in A549 human lung adenocarcinoma cell line. Prostaglandins Leukot Essent Fatty Acids.

[20] Huynh H, Nguyen TT, Chan E, Tran E (2003). Inhibition of *ErbB-2* and *ErbB-3* expression by quercetin prevents transforming growth factor alpha (tgf-α)– and epidermal growth factor (egf)–induced human pc-3 prostate cancer cell proliferation. Int J Oncol.

[21] Ma ZS, Huynh TH, Ng CP, Do PT, Nguyen TH, Huynh H (2004). Reduction of CWR22 prostate tumor xenograft growth by combined tamoxifen–quercetin treatment is associated with inhibition of angiogenesis and cellular proliferation. Int J Oncol.

[22] Slamon DJ, Clark GM, Wong SG, Levin WJ, Ullrich A, McGuire WL (1987). Human breast cancer: correlation of relapse and survival with amplification of the *HER-2*/*neu* oncogene. Science.

[23] Slamon DJ, Godolphin W, Jones LA (1989). Studies of the *HER-2*/*neu* proto-oncogene in human breast and ovarian cancer. Science.

[24] Allgayer H, Babic R, Gruetzner KU, Tarabichi A, Schildberg FW, Heiss MM (2000). c-*ErbB-*2 is of independent prognostic relevance in gastric cancer and is associated with the expression of tumor-associated protease systems. J Clin Oncol.

[25] Slamon DJ, Leyland–Jones B, Shak S (2001). Use of chemotherapy plus a monoclonal antibody against *HER2* for meta-static breast cancer that overexpresses *HER2.*. N Engl J Med.

[26] Agus DB, Akita RW, Fox WD (2002). Targeting ligand-activated *ErbB2* signaling inhibits breast and prostate tumor growth. Cancer Cell.

[27] Blackwell KL, Dewhirst MW, Liotcheva V (2004). *HER-2* gene amplification correlates with higher levels of angiogenesis and lower levels of hypoxia in primary breast tumors. Clin Cancer Res.

[28] Menendez JA, Vellon L, Colomer R, Lupu R (2005). Oleic acid, the main monounsaturated fatty acid of olive oil, suppresses *HER-2*/*neu* (*ErbB*-2) expression and synergistically enhances the growth inhibitory effects of trastuzumab (Herceptin) in breast cancer cells with *HER-2*/*neu* oncogene amplification. Ann Oncol.

[29] Wasserman L, Avigad S, Beery E, Nordenberg J, Fenig E (2002). The effect of aloe emodin on the proliferation of a new Merkel carcinoma cell line. Am J Dermatopathol.

[30] Davies G, Salter J, Hills M, Martin LA, Sacks N, Dowsett M (2003). Correlation between cyclooxygenase-2 expression and angiogenesis in human breast cancer. Clin Cancer Res.

[31] Ben Ezra D (1978). Neovasculogenic ability of prostaglandins, growth factors and synthetic chemoattractants. Am J Ophthalmol.

[32] Ziche M, Jones J, Gullino PM (1982). Role of prostaglandin E1 and copper in angiogenesis. J Nat Cancer Inst.

[33] Form DM, Auerbach R (1983). pge2 and angiogenesis. Proc Soc Exp Biol Med.

[34] Gately S, Li WW (2004). Multiple roles of cox-2 in tumor angiogenesis: a target for antiangiogenic therapy. Semin Oncol.

[35] Ruegg C, Dormond O, Mariotti A (2004). Endothelial cell integrins and cox-2: mediators and therapeutic targets of tumor angiogenesis. Biochim Biophys Acta.

[36] Gately S, Kerbel R (2003). Therapeutic potential of selective cyclooxygenase-2 inhibitors in the management of tumor angiogenesis. Prog Exp Tumor Res.

[37] Connolly JM, Liu XH, Rose DP (1996). Dietary linoleic acid–stimulated human breast cancer cell growth and metastasis in nude mice and their suppression by indomethacin, a cyclooxygenase inhibitor. Nutr Cancer.

[38] Rose DP, Connolly JM, Coleman M (1996). Effect of N-3 fatty acids on the progression of metastases after the surgical excision of human breast cancer cell solid tumors growing in nude mice. Clin Cancer Res.

[39] Rose DP, Connolly JM (1999). Omega-3 fatty acids as cancer chemopreventive agents. Pharmacol Ther.

[40] Connolly JM, Liu XH, Rose DP (1997). Effects of dietary menhaden oil, soy, and a cyclooxygenase inhibitor on human breast cancer cell growth and metastasis in nude mice. Nutr Cancer.

[41] Larsson SC, Kumlin M, Ingelman–Sundberg M, Wolk A (2004). Dietary long-chain N-3 fatty acids for the prevention of cancer: a review of potential mechanisms. Am J Clin Nutr.

[42] Wang ZY, Nixon DW (2001). Licorice and cancer. Nutr Cancer.

[43] Shibata A, Nagaya T, Imai T, Funahashi H, Nakao A, Seo H (2002). Inhibition of nf-κb activity decreases the vegf mrna expression in MDA-MB-231 breast cancer cells. Breast Cancer Res Treat.

[44] Yu HG, Zhong X, Yang YN (2004). Increased expression of nuclear factor-κB/RelA is correlated with tumor angiogenesis in human colorectal cancer. Int J Colorectal Dis.

[45] Sunwoo JB, Chen Z, Dong G (2001). Novel proteasome inhibitor PS-341 inhibits activation of nuclear factor-κB, cell survival, tumor growth, and angiogenesis in squamous cell carcinoma. Clin Cancer Res.

[46] Shishodia S, Koul D, Aggarwal BB (2004). Cyclooxygenase (cox)–2 inhibitor celecoxib abrogates tnf-induced nf-κb activation through inhibition of activation of I κ B alpha kinase and *akt* in human non-small cell lung carcinoma: correlation with suppression of cox-2 synthesis. J Immunol.

[47] Guinebretiere JM, Le Monique G, Gavoille A, Bahi J, Contesso G (1994). Angiogenesis and risk of breast cancer in women with fibrocystic disease. J Nat Cancer Inst.

[48] Fregene TA, Kellogg CM, Pienta KJ (1994). Microvessel quantification as a measure of angiogenic activity in benign breast tissue lesions: marker for precancerous disease?. Int J Oncol.

[49] Heffelfinger SC, Yassin R, Miller MA, Lower E (1996). Vascularity of proliferative breast disease and carcinoma *in situ* correlates with histological features. Clin Cancer Res.

[50] Brawer MK, Deering RE, Brown M, Preston SD, Bigler SA (1994). Predictors of pathologic stage in prostatic carcinoma. The role of neovascularity. Cancer.

[51] Subbaramaiah K, Dannenberg AJ (2003). Cyclooxygenase-2: a molecular target for chemoprevention and treatment. Trends Pharmacol Sci.

[52] Iniguez MA, Rodriguez A, Volpert OV, Fresno M, Redondo JM (2003). Cyclooxygenase-2: a therapeutic target for angiogenesis. Trends Mol Med.

[53] Plummer SM, Holloway KA, Manson MM (1999). Inhibition of cyclooxygenase-2 expression in colon cells by the chemopreventive agent curcumin involves inhibition of nf-κb activation via the *NIK*/*IKK* signalling complex. Oncogene.

[54] Subbaramaiah K, Chung WJ, Michaluart P (1998). Resveratrol inhibits cyclooxygenase-2 transcription and activity in phorbol ester-treated human mammary epithelial cells. J Biol Chem.

[55] Oh GS, Pae HO, Choi BM (2004). 20(*S*)-Protopanaxatriol, one of ginsenoside metabolites, inhibits inducible nitric oxide synthase and cyclooxygenase-2 expressions through inactivation of nuclear factor-κB in RAW 264.7 macrophages stimulated with lipopolysaccharide. Cancer Lett.

[56] Bode AM, Ma WY, Surh YJ, Dong Z (2001). Inhibition of epidermal growth factor induced cell transformation and AP1 activation by [6]-gingerol. Cancer Res.

[57] Wallace JM (2002). Nutritional and botanical modulation of the inflammatory cascade: eicosanoids, cyclooxygenases, and lipoxygenases as an adjunct in cancer therapy. Integr Cancer Ther.

[58] Pendurthi UR, Williams JT, Rao LV (1997). Inhibition of tissue factor gene activation in cultured endothelial cells by curcumin. Suppression of activation of transcription factors Egr-1, ap-1, and nf-κ b. Arterioscler Thromb Vasc Biol.

[59] Jobin C, Bradham CA, Russo MP (1999). Curcumin blocks cytokine-mediated nf-κ b activation and proinflammatory gene expression by inhibiting inhibitory factor i-κ b kinase activity. J Immunol.

[60] Bharti AC, Donato N, Singh S, Aggarwal BB (2003). Curcumin (diferuloylmethane) down-regulates the constitutive activation of nuclear factor-κ b and IκBα kinase in human multiple myeloma cells, leading to suppression of proliferation and induction of apoptosis. Blood.

[61] Siwak DR, Shishodia S, Aggarwal BB, Kurzrock R (2005). Curcumin-induced antiproliferative and proapoptotic effects in melanoma cells are associated with suppression of IκB kinase and nuclear factor κb activity and are independent of the B-*raf*/mitogen–activated/extracellular signal-regulated protein kinase pathway and the *akt* pathway. Cancer.

[62] Surh YJ, Na HK, Lee SS (2004). Transcription factors and mitogen-activated protein kinases as molecular targets for chemo-prevention with anti-inflammatory phytochemicals. Biofactors.

[63] Huang P, Oliff A (2001). Signaling pathways in apoptosis as potential targets for cancer therapy. Trends Cell Biol.

[64] Roberts WG, Whalen PM, Soderstrom E (2005). Antiangiogenic and antitumor activity of a selective pdgfr tyrosine kinase inhibitor, CP-673, 451. Cancer.

[65] Jiang BH, Zheng JZ, Aoki M, Vogt PK (2000). Phosphatidylinositol 3-kinase signaling mediates angiogenesis and expression of vascular endothelial growth factor in endothelial cells. Proc Natl Acad Sci U S A.

[66] Amin MA, Volpert OV, Woods JM, Kumar P, Harlow LA, Koch AE (2003). Migration inhibitory factor mediates angiogenesis via mitogen-activated protein kinase and phosphatidylinositol kinase. Circ Res.

[67] Haspel HC, Scicli GM, McMahon G, Scicli AG (2002). Inhibition of vascular endothelial growth factor-associated tyrosine kinase activity with SU5416 blocks sprouting in the microvascular endothelial cell spheroid model of angiogenesis. Microvasc Res.

[68] Bold G, Altmann KH, Frei J (2000). New anilinophthalazines as potent and orally well absorbed inhibitors of the vegf receptor tyrosine kinases useful as antagonists of tumor-driven angiogenesis. J Med Chem.

[69] Wang Y, Wei X, Xiao X (2005). Arachidonic acid epoxygenase metabolites stimulate endothelial cell growth and angiogenesis via mitogen-activated protein kinase and phosphatidylinositol 3-kinase/*akt* signaling pathways. J Pharmacol Exp Ther.

[70] Lauthier F, Taillet L, Trouillas P, Delage C, Simon A (2000). Ursolic acid triggers calcium-dependent apoptosis in human Daudi cells. Anticancer Drugs.

[71] Danilenko M, Wang X, Studzinski GP (2001). Carnosic acid and promotion of monocytic differentiation of HL60-G cells initiated by other agents. J Natl Cancer Inst.

[72] Plouzek CA, Ciolino HP, Clarke R, Yeh GC (1999). Inhibition of P-glycoprotein activity and reversal of multidrug resistance *in vitro* by rosemary extract. Eur J Cancer.

[73] Dorrie J, Sapala K, Zunino SJ (2001). Carnosol-induced apoptosis and downregulation of Bcl-2 in B-lineage leukemia cells. Cancer Lett.

[74] Ren MQ, Kuhn G, Wegner J, Chen J (2001). Isoflavones, substances with multi-biological and clinical properties. Eur J Nutr.

[75] Brownson DM, Azios NG, Fuqua BK, Dharmawardhane SF, Mabry TJ (2002). Flavonoid effects relevant to cancer. J Nutr.

[76] Sachinidis A, Hescheler J (2002). Are catechins natural tyrosine kinase inhibitors?. Drug News Perspect.

[77] Lin YL, Liang YC, Lee SS, Chiang BL (2005). Polysaccharide purified from *Ganoderma lucidum* induced activation and maturation of human monocyte-derived dendritic cells by the nf–κb and p38 mitogen-activated protein kinase pathways. J Leukoc Biol.

[78] Adams JM, Cory S (1998). The Bcl-2 protein family: arbiters of cell survival. Science.

[79] Cheng EH, Wei MC, Weiler S (2001). Bcl-2, Bcl-XL, sequester BH3 domain-only molecules preventing *BAX*-and *BAK*-mediated mitochondrial apoptosis. Mol Cell.

[80] Saito M, Korsmeyer SJ, Schlesinger PH (2000). *BAX*-dependent transport of cytochrome C reconstituted in pure liposomes. Nature Cell Biology.

[81] Yang E, Zha J, Jockel J, Boise LH, Thompson CB, Korsmeyer SJ (1995). *BAD*, a heterodimeric partner for Bcl-XL and Bcl-2, displaces *BAX* and promotes cell death. Cell.

[82] Fontanini G, Boldrini L, Vignati S (1998). *BCL2* and *p53* regulate vascular endothelial growth factor (vegf)-mediated angiogenesis in non-small cell lung cancer. Eur J Cancer.

[83] Choudhuri T, Pal S, Das T, Sa G (2005). Curcumin selectively induces apoptosis in deregulated cyclin D1–expressed cells at G2 phase of cell cycle in a *p53-*dependent manner. J Biol Chem.

[84] Kuo ML, Huang TS, Lin JK (1996). Curcumin, an antioxidant and anti-tumor promoter, induces apoptosis in human leukemia cells. Biochim Biophys Acta.

[85] Leone M, Zhai D, Sareth S, Kitada S, Reed JC, Pellecchia M (2003). Cancer prevention by tea polyphenols is linked to their direct inhibition of antiapoptotic Bcl-2–family proteins. Cancer Res.

[86] Chen Y, Yang L, Lee TJ (2000). Oroxylin A inhibition of lipopolysac-charide-induced *INOS* and *COX-2* gene expression via suppression of nuclear factor-κb activation. Biochem Pharmacol.

[87] Powell CB, Fung P, Jackson J (2003). Aqueous extract of herba *Scutellaria barbatae*, a Chinese herb used for ovarian cancer, induces apoptosis of ovarian cancer cell lines. Gynecol Oncol.

[88] Tseng TH, Kao TW, Chu CY, Chou FP, Lin WL, Wang CJ (2000). Induction of apoptosis by hibiscus protocatechuic acid in human leukemia cells via reduction of retinoblastoma (rb) phosphorylation and *BCL-2* expression. Biochem Pharmacol.

[89] Tseng TH, Hsu JD, Lo MH (1998). Inhibitory effect of *Hibiscus* protocatechuic acid on tumor promotion in mouse skin. Cancer Lett.

[90] Hong C, Firestone GL, Bjeldanes LF (2002). *BCL-2* family-mediated apoptotic effects of 3,3′-diindolylmethane (dim) in human breast cancer cells. Biochem Pharmacol.

[91] Choi SH, Lyu SY, Park WB (2004). Mistletoe lectin induces apoptosis and telomerase inhibition in human A253 cancer cells through dephosphorylation of *akt.*. Arch Pharm Res.

[92] Wang CC, Chen LG, Lee LT, Yang LL (2003). Effects of 6-gingerol, an antioxidant from ginger, on inducing apoptosis in human leukemic HL-60 cells. In vivo.

[93] Bagchi D, Bagchi M, Stohs SJ (2000). Free radicals and grape seed proanthocyanidin extract: importance in human health and disease prevention. Toxicology.

[94] Tong X, Lin S, Fujii M, Hou DX (2004). Molecular mechanisms of echinocystic acid-induced apoptosis in HepG2 cells. Biochem Biophys Res Commun.

[95] Tong X, Lin S, Fujii M, Hou DX (2004). Echinocystic acid induces apoptosis in HL-60 cells through mitochondria-mediated death pathway. Cancer Lett.

[96] Zhang S, Ong CN, Shen HM (2004). Involvement of proapoptotic *BCL-2* family members in parthenolide-induced mitochondrial dysfunction and apoptosis. Cancer Lett.

[97] Woo HJ, Choi YH (2005). Growth inhibition of A549 human lung carcinoma cells by beta-lapachone through induction of apoptosis and inhibition of telomerase activity. Int J Oncol.

[98] Lee JH, Cheong J, Park YM, Choi YH (2005). Down-regulation of cyclooxygenase-2 and telomerase activity by beta-lapachone in human prostate carcinoma cells. Pharmacol Res.

[99] Park DI, Lee JH, Moon SK (2005). Induction of apoptosis and inhibition of telomerase activity by aqueous extract from *Platycodon grandiflorum* in human lung carcinoma cells. Pharmacol Res.

[100] Hejna M, Raderer M, Zielinski CC (1999). Inhibition of metastases by anticoagulants. J Natl Cancer Inst.

[101] Smorenburg SM, Van Noorden CJ (2001). The complex effects of heparins on cancer progression and metastasis in experimental studies. Pharmacol Rev.

[102] Blom JW, Doggen CJ, Osanto S, Rosendaal FR (2005). Malignancies, prothrombotic mutations, and the risk of venous thrombosis. JAMA.

[103] Wang S, Zheng Z, Weng Y (2004). Angiogenesis and anti-angiogenesis activity of Chinese medicinal herbal extracts. Life Sci.

[104] Samuels N (2005). Herbal remedies and anticoagulant therapy. Thromb Haemost.

[105] Huang GW, Xie CX, Kuang GQ (2003). Treatment of 41 patients with advanced stage of nasopharyngeal cancer by combination therapy of radiation and Chinese herbal drugs for activating blood circulation to remove stasis as hirudo [Chinese]. Zhongguo Zhong Xi Yi Jie He Za Ahi.

[106] Xu GZ, Cai WM, Qin DX (1989). Chinese herb “destagnation” series i: Combination of radiation with destagnation in the treatment of nasopharyngeal carcinoma: a prospective randomized trial on 188 cases. Int J Radiat Oncol Biol Phys.

[107] Jung YD, Ahmad SA, Akagi Y (2000). Role of the tumor microenvironment in mediating response to anti-angiogenic therapy. Cancer Metastasis Rev.

[108] Sweeney CJ, Miller KDA, Sledge GW (2003). Resistance in the anti-angiogenic era: nay-saying or a word of caution?. Trends Mol Med.

[109] Ria R, Portaluri M, Russo F (2004). Serum levels of angiogenic cytokines decrease after antineoplastic radiotherapy. Cancer Lett.

[110] Yoshida S, Ono M, Shono T (1997). Involvement of interleukin-8, vascular endothelial growth factor, and basic fibroblast growth factor in tumor necrosis factor-α dependent angiogenesis. Mol Cell Biol.

[111] Tang FY, Meydani M (2001). Green tea catechins and vitamin E inhibit angiogenesis of human microvascular endothelial cells through suppression of IL-8 production. Nutr Cancer.

[112] Brower V (2003). Evidence of efficacy: researchers investigating markers for angiogenesis inhibitors. J Natl Cancer Inst.

[113] Ruegg C, Meuwly JY, Driscoll R, Werffeli P, Zaman K, Stupp R (2003). The quest for surrogate markers of angiogenesis: a paradigm for translational research in tumor angiogenesis and antiangiogenesis trials. Curr Mol Med.

[114] Salcedo X, Medina J, Sanz–Cameno P, Garcia–Buey L, Martin–Vilchez S, Moreno–Otero R (2005). Review article: angiogenesis soluble factors as liver disease markers. Aliment Pharmacol Ther.

[115] Schneider M, Tjwa M, Carmeliet P (2005). A surrogate marker to monitor angiogenesis at last. Cancer Cell.

[116] Shaked Y, Bertolini F, Man S (2005). Genetic heterogeneity of the vasculogenic phenotype parallels angiogenesis; implications for cellular surrogate marker analysis of antiangiogenesis. Cancer Cell.

[117] Neeman M (2000). Preclinical mri experience in imaging angiogenesis. Cancer Metastasis Rev.

[118] AE 941 (2004). Drugs R D.

